# Solid lipid nanoparticle delivery of rhynchophylline enhanced the efficiency of allergic asthma treatment via the upregulation of suppressor of cytokine signaling 1 by repressing the p38 signaling pathway

**DOI:** 10.1080/21655979.2021.1988364

**Published:** 2021-10-21

**Authors:** Chuanfeng Lv, Hui Li, Hongxia Cui, Qianyu Bi, Meng Wang

**Affiliations:** a Department of Pharmacology, Jining No.1 People’s Hospital, Jining, Shandong, China; b Department of Medical Affairs, Jining No.1 People’s Hospital, Jining, Shandong, China; c Department of Respiratory Oncology, Jining No.1 People’s Hospital, Jining, Shandong, China; d College of Traditional Chinese Medicine, Shandong University of Traditional Chinese Medicine, Jinan, Shandong, China

**Keywords:** Rhynchophylline, solid lipid nanoparticle, allergic asthma, suppressor of cytokine signaling 1, p38 signaling pathway

## Abstract

Allergic asthma is one of the most common chronic airway diseases, and there is still a lack of effective drugs for the treatment of allergic asthma. The purpose of this work is to formulate rhynchophylline (Rhy)-solid lipid nanoparticles (SLNs) to improve their therapeutic efficacy in a mice allergic model of asthma. A solvent injection method was employed to prepare the Rhy-SLNs. Physicochemical characterization of Rhy-SLNs was measured, and the release assessment was investigated, followed by the release kinetics. Next, a model of murine experimental asthma was established. Mice were subcutaneously injected with 20 μg ovalbumin mixed with 1 mg aluminum hydroxide on days 0, 14, 28, and 42 and administrated aerosolized 1% ovalbumin (w/v) by inhalation from day 21 to day 42. Mice were intraperitoneally injected with 20 mg/kg Rhy-SLNs or Rhy at one hour before the airway challenge with ovalbumin. The results showed that Rhy-SLNs revealed a mean particle size of 62.06 ± 1.62 nm with a zeta potential value of −6.53 ± 0.04 mV and 82.6 ± 1.8% drug entrapment efficiency. The release curve of Rhy-SLNs was much higher than the drug released in phosphate buffer saline at 0, 1, 1.5, 2, 4, or 6 h. Moreover, Rhy-SLNs exerted better effects on inhibiting ovalbumin-induced airway inflammation, oxidative stress, airway remodeling (including collagen deposition and mucus gland hyperplasia) than Rhy in murine experimental asthma. Subsequently, we found that Rhy-SLNs relieved allergic asthma via the upregulation of the suppressor of cytokine signaling 1 by repressing the p38 signaling pathway.

## Introduction

Asthma, a chronic airway disease, affects approximately 300 million people worldwide [1]. Asthma can be divided into non-allergic and allergic, which is the most common asthma phenotype [2]. Clinically, allergic asthma presents a typical constellation of symptoms, such as, chest tightness, wheezing, coughing, and shortness of breath, which could be controlled but not cured [3]. These clinical symptoms are resulted from multicellular inflammation, airway hyperresponsiveness, airway remodeling, and oxidative stress of allergic asthma [4]. The airways of asthmatics usually present with the infiltration of inflammatory cells (neutrophil, eosinophil, lymphocyte, and macrophage) and exhibit high levels of inflammatory cytokines (interleukin (IL)-4, IL-5, and IL-13). These cytokines further stimulate the production of IgE [5]. The prolonged inflammatory process of asthma causes the production of reactive oxygen species (ROS) [6]. Long-term exposure to ROS induces the oxidative stress of airway, which might result in airway hyperresponsiveness and bronchoconstriction [6]. It has been reported that the weakened activities of antioxidants contribute to the development of asthma [7]. Moreover, the persistent chronic inflammation of allergic asthma triggers the inflammation of lung tissues and airway walls, leading to the structural changes and bronchoconstriction, which are recognized as airway remodeling [8]. Goblet gland hyperplasia and collagen deposition are regarded as features of airway remodeling in severe asthma [9]. Currently, inhaled corticosteroids are widely used for the treatment of asthma. Approximately 10–40% of the inhaled corticosteroids are deposited in the lungs, while the rest of them are swallowed. Then, this swallowed corticosteroid enters the gastrointestinal tract in which, during a prolonged period, it can have undesired local effects that seriously affect the life quality of the asthmatics [10,11]. Thus, there is an urgent need to explore novel drugs for allergic asthma therapy.

*Uncaria rhynchophylla*, a traditional Chinese herb, has been used for the treatment on central nervous system and cardiovascular diseases for a long time [12]. Rhynchophylline (Rhy) is the major active ingredient of *Uncaria rhynchophylla* and possesses anti-inflammatory, vasodilatory, and anti-hypertensive functions [13–15]. In our previous study, we have found that Rhy markedly attenuates the allergic bronchial asthma, as evidenced by the alleviation of airway inflammation, airway remodeling, and oxidative stress [16]. The protective role of Rhy in asthma is mediated by repressing the smad and mitogen-activated protein kinase (MAPK) signaling transductions [17]. These results suggest the potential of Rhy as a novel anti-asthma drug. However, the application of Rhy is limited due to its low bioavailability and low aqueous solubility [18]. Looking for a drug carrier system for Rhy that can solve these problems and improve its therapeutic efficiency might be a great policy for asthma treatment.

Solid lipid nanoparticles (SLNs) are a new drug delivery system and are composed of solid natural or synthetic lipid as carrier and drug encapsulated in lipid nucleus [19]. It can not only improve the solubility of drugs in nano-formulations but also their bioavailability [19]. Wang et al. [20] have reported that administration of curcumin-SLNs is more effective than curcumin to mitigate the progression of asthma. Castellani et al. [21] have proven that proanthocyanidins loaded in SLNs allow them to serve as a better role in suppressing the oxidant stress of airway epithelial cells. Proanthocyanidins-SLNs are characterized by stability and long-term persistence. These studies indicate the potential of SLNs on enhancing the drug therapeutic efficacy as a drug delivery carrier.

Based on the anti-asthma function of Rhy as we previously described, we speculated that encapsulation of Rhy with SLNs might promote the inhibitory effects of Rhy on asthma. The current study aimed to formulate Rhy-SLNs to improve their therapeutic efficacy in an ovalbumin (OVA)-induced mice allergic model of asthma. Herein, we explored the physiochemical characterization of Rhy-SLNs and compared the suppressed effects of Rhy-SLNs/Rhy on OVA-induced airway inflammation, oxidative stress, airway remodeling of murine in experimental asthma. The underlying mechanism was further investigated.

## Material and methods

### Ethics approval

The animal experimental protocols were approved by The Medical Ethic Committee of Jining No.1 People’s Hospital following The Guideline for the Care and Use of Laboratory Animals.

### Preparation of Rhy-SLNs

Rhy-SLNs were prepared as per the solvent injection method described previously [22]. Briefly, 50 mg glyceryl monostearate, 50 mg Tween-80, and 100 mg Solutol HS 15 were dissolved at 80°C as organic phase. 2 mg Rhy was further dissolved in the organic phase. Next, an aqueous phase was prepared by heating 10 mL distilled water to 80°C. The aqueous phase was added to the hot organic phase under mechanical agitation at 2000 rpm for 5 min. Subsequently, the resultant emulsions were cooled to room temperature while stirring to form Rhy-SLNs. Physical characterization using transmission electron microscope, differential scanning calorimetry and X-ray diffraction (data was not shown) confirmed the interaction between Rhy and SLNs corroborating that the drug was efficiently encapsulated by SLNs.

### The quantification of entrapment efficiency of Rhy-SLNs

The entrapment efficiency of Rhy in SLNs was evaluated as previously described [23,24], and the parameters were detected by Agilent 1260 infinity high performance liquid chromatography (HPLC; Agilent Technologies, Santa Clara, CA, USA). In detail, 0.5 mL Rhy-SLNs was diluted with ultrapure water to 3 mL. The mixture was introduced to an ultrafiltration tube (Millipore, Billerica, MA, USA), followed by a centrifugation at 3000 rpm for 5 min. The supernatant was determined by HPLC to detect the amount of free drug (W_free_). Subsequently, 0.5 mL Rhy-SLNs was diluted with ethanol to 3 mL. The mixture was ultrasonicated for 5 min and then underwent the same procedure described above to detect the total amount of Rhy (W_total_). Chromatographic conditions were listed as follows: mobile phase, methanol/0.02% triethylamine (7:3, v/v); flow rate, 1 mL/min; column temperature, 30°C; sample volume, 20 μL; detected wavelength, 244 nm. The entrapment efficiency was calculated as follows: (W_total_ – W_free_)/W_total_ * 100%. The drug loading was calculated as follows: (W_total_ – W_free_)/W_lipids_ * 100%. W_lipids_ was the total amount of lipids.

### The morphology, particle size, and zeta potential assessment

The morphology of Rhy-SLNs and SLNs were evaluated by transmission electron microscope with JEOL.JEM-1200EXII electron microscope (Japan Electron Optics Laboratory, Tokyo, Japan). The mean particle size, polymer dispersity index, and zeta potential value of Rhy-SLNs were assessed via photon correlation spectroscopy by Zetasizer Nano-ZS mastersizer (Malvern Instruments, Malvern, UK). The value was measured in triplicate.

### Rhy-SLNs/Rhy release and release kinetics

The release study for Rhy and Rhy-SLNs was performed by using the dialysis membrane method as previously described [23]. Briefly, 500 μg Rhy-SLNs dissolved in 2.5 mL phosphate buffer saline (PBS, pH = 7.4) within 0.2% tween-80 (v/v) were transferred to a dialysis bag. The bag was introduced into a conical flask with stoppers containing 75 mL PBS with 0.2% tween-80 (v/v) in a shaker with 50 rpm at 37°C. At pre-determined time intervals (0.5, 1, 1.5, 2, 4, and 6 h), an aliquot (1.5 mL) of dialysis medium was replaced with the same amount of fresh medium. Rhy underwent the same procedure explained above. The Rhy concentration in the released samples was detected by the HPLC system described above.

Then, the zero-order model, the first-order model, the Higuchi model, the Ritger-Peppas model, and the Weibull model were used to model the Rhy and Rhy-SLNs release kinetics as previously described [25,26]. Zero-order model: Q_0 –_ Q_t_ = kt; First-order model: lnQ_0 –_ lnQ_t_ = kt; Higuchi model: Q_0 –_ Q_t_ = kt^1/2^; Ritger-Peppas: Q_0 –_ Q_t_ = kt^n^; Weibull model: Q_0_ – Q_t_ = 1 – exp(-kt^β^). Q_0_ and Q_t_ are the remained amounts of drug in the time zero and t, respectively. K is the rate constant, and β is the release exponent. The squared correlation coefficient (R^2^) was used to determine the most appropriate drug release model by comparison of experimental data to predicted models.

### OVA mouse model and Rhy-SLNs application

**Figure 1.**
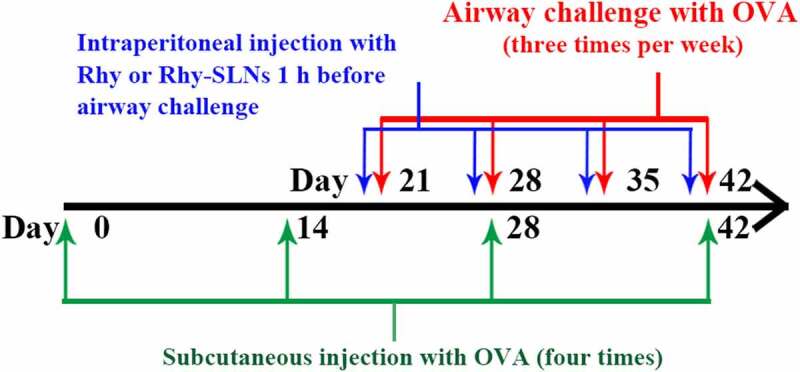
A schematic overview of asthma induction in mice. OVA, ovalbumin; Rhy, rhynchophylline; Rhy-SLNs, rhynchophylline-solid lipid nanoparticles

The OVA mouse model was established according to the method described previously [17]. Eight-week-old female BALB/c mice were purchased from Liaoning Changsheng biotechnology (Benxi, China) and placed in a 12 h light-dark cycle at 22 ± 1°C with 45–55% humidity and with food and water ad libitum. A total of thirty mice were randomly divided into five groups (six mice per group: control, OVA, OVA+Blank-SLNs, OVA+Rhy-SLNs, and OVA+Rhy) after 1 week of acclimation. For the establishment of asthma model, mice were sensitized and made allergic to 20 μg OVA mixed with 1 mg aluminum hydroxide via subcutaneous injection on days 0, 14, 28, and 42 of the model induction. From day 21 to day 42 of the induction, mice were administrated aerosolized 1% OVA (w/v) by inhalation for 30 min, three times a week. Mice in the control group underwent the same procedure described above, but PBS instead of the OVA. For drug treatment, mice were intraperitoneally injected with 20 mg/kg Rhy-SLNs or 20 mg/kg Rhy at 1 hour before the airway challenge with OVA. Mice in OVA+Blank-SLNs group received an equal amount of Blank-SLNs, and mice in the control and OVA groups were administered with the same volume of solvent. A schematic overview of asthma induction in mice is shown in Figure 1. Mice that exhibited the characteristics of allergic asthma including inflammation, oxidative stress, and airway remodeling after OVA induction were regarded as the murine experimental asthma model. Mice were sacrificed 24 h after the last airway challenge with OVA. The lung tissues, bronchoalveolar lavage fluid (BALF), and serum were collected for the subsequent experiments.

### BALF

BALF was obtained according to the method described previously [27]. BALF was harvested 24 h after the last airway challenge with OVA. Mice sacrificed, and the tracheas were cannulated. 0.8 mL ice-cold PBS was instilled into the lung tissues for three times. The total number of cells was counted. Then, the BALF was centrifugated at 300 × g for 10 min to obtain the cell pellets, which were further resuspended in PBS. Next, cells were subjected to Wright-Giemsa staining, followed by the cell counts. Cells were classified as neutrophil, lymphocyte, eosinophil, and macrophage.

### Enzyme-linked immunosorbent assays (ELISA)

The concentration of IgE, IL-4, IL-5, and IL-13 was detected by using ELISA [28]. Serum was collected 24 h after the last airway challenge with OVA. After centrifugation at 1000 × g for 10 min, the concentration of IgE, IL-4, IL-5, and IL-13 was detected by using Mouse ELISA Kits (IgE, IL-4, IL-5, and IL-13; MultiScience (Lianke) Biotech, Hangzhou, China) as per the manufacturers’ instructions.

### The detection of ROS

The content of ROS was evaluated as previously described [29]. After rinsed with distilled water for three times, frozen sections of lung tissues (10 μm) were incubated with ROS probe solution (Bestbio, Shanghai, China) at 37°C preventing from the light for 30 min and then washed with PBS for 3 min. Next, sections were blocked with antifade solution (Solarbio), and the images were captured by BX53 microscope (Olympus, Tokyo, Japan).

### The quantification of superoxide dismutase (SOD) and glutathione (GSH)

The content of SOD and GSH was quantified as previously described [30]. The lung tissues were mixed with normal saline (g/v = 1:9) and then mechanically agitated in ice-cold water bath. After centrifugation at 2500 rpm for 10 min, the supernatant was used for the measurement. The protein concentration was detected via employing a Bicinchoninic Acid Protein Assay Kit (Beyotime, Shanghai, China). The quantification of SOD and GSH was performed by a SOD assay kit and a GSH assay kit following the users’ protocols. All the kits were purchased from Nanjing Jiancheng Bioengineering Institute (Nanjing, China).

### Histologic examination

Hematoxylin-eosin staining was carried out to determine the infiltration of inflammatory cells, which reflected the airway inflammation of murine experimental asthma [31]. Paraffin-embedded sections of lung tissue (5 μm) were deparaffinized in xylene (Aladdin, Shanghai, China) and rehydrated in an ethanol gradient with distilled water. Then, sections were stained with hematoxylin (Sinopharm, Beijing, China) for 5 min and eosin (Sinopharm) for 3 min. The images were captured with BX53 microscopy. To assess the severity of inflammatory infiltration, peribronchial cell counts were carried out based on the following grading scale: 0, no inflammatory cells; 1, a few inflammatory cells; 2, a ring of inflammatory cells one cell layer deep; 3, a ring of inflammatory cells two to four cells deep; 4, a ring of inflammatory cells more than four cells deep [31]. The higher score, the severe inflammatory infiltration.

Masson’s trichrome staining was used to examine the severity of collagen deposition in lung tissues [32]. Paraffin-embedded sections of lung tissues (5 μm) were deparaffinized in xylene and rehydrated in an ethanol gradient with distilled water. Next, sections were stained with regaud hematoxylin (Sinopharm) for 6 min and then stained with li chunhong acid fuchsin solution (Sinopharm) for 1 min. After washed with 0.2% glacial acetic acid, a drop of 1% phosphomolybdic acid (Sinopharm) was added to the sections for 5 min, and the section was further counterstained with aniline blue for 5 min. The images were captured with BX53 microscopy.

Periodic acid-Schiff staining was used to evaluate the severity of goblet cell hyperplasia of lung tissues [33]. Paraffin-embedded sections of lung tissues (5 μm) were deparaffinized in xylene and rehydrated in an ethanol gradient with distilled water. Then, periodic acid was added dropwise to the sections for 10 min, followed by staining with schiff’s reagent (Beijing leagene biotech, Beijing, China) for 15 min. After washed with distilled water, sections were counterstained with hematoxylin for 2 min. The images were captured with BX53 microscopy. To evaluate the content of mucus production, mucus gland hyperplasia was quantified by the following grading scale: 0, no goblet cells; 1, less than 25% of goblet cells; 2, between 25% and 50% of goblet cells; 3, between 50% and 75% of goblet cells; 4, more than 75% of goblet cells [33]. The higher score, the more mucus production.

### Western blot

Total protein was extracted from lung tissues exposed to OVA by lysis solution (mixed with 1% phenylmethylsulfonyl fluoride) and was quantified by using a Bicinchoninic Acid Protein Assay Kit (Solarbio, Beijing, China). 20 μg protein was separated by sodium dodecyl sulfate polyacrylamide gel electrophoresis followed by a transfer to polyvinylidene fluoride membranes (Millipore). Next, membranes were blocked with 5% (M/V) skimmed milk for an hour and were incubated with primary antibodies including alpha-smooth muscle actin (α-SMA; Affinity, Changzhou, China), collagen I (Affinity), suppressor of cytokine signaling 1 (SOCS1; ABclonal Biotechnology, Shanghai, China), p38 (ABclonal Biotechnology), and p-p38 (ABclonal Biotechnology) at 4°C overnight. Glyceraldehyde-3-phosphate dehydrogenase (Proteintech. Wuhan, China) was used as a loading control. All primary antibodies were diluted 1:1000 except for glyceraldehyde-3-phosphate dehydrogenase (diluted 1:10,000). Subsequently, protein blots were incubated with horseradish peroxidase-conjugated secondary antibodies (diluted 1:3000; Solarbio) at 37°C for an hour. The relative protein levels were visualized by employing horseradish peroxidase chromogenic substrate (Solarbio). The images were captured by WD-9413B gel imaging system (Liuyi Biotechnology, Beijing, China).

### Statistical analysis

Data was expressed as mean ± standard deviation except for the boxplot (median, interquartile range, maximum, and minimum of separate repeats) (n = 6 for in vivo experiments; n = 3 for in vitro experiments). The selected sample size was larger than that of predicted by power analysis using G*power 3.1.9.7 software (30 > 25; power = 0.8, α err probe = 0.05). Statistical evaluation of data was performed by one-way analysis of variance except for drug release (two-way analysis of variance). Inflammatory score and mucus score were analyzed by non-parametric analysis (Kruskal–Wallis test). Statistically significant difference was assumed when p < 0.05.

## Results

Allergic asthma is one of the most common chronic airway diseases, and there is still a lack of effective drugs for the treatment of allergic asthma. Based on the anti-asthma function of Rhy as we previously described, we speculated that encapsulation of Rhy with SLNs might promote the inhibitory effects of Rhy on asthma. The current study aimed to formulate Rhy-SLNs to improve their therapeutic efficacy in an OVA-induced mice allergic model of asthma. The results showed that Rhy-SLNs exerted better effects on repressing airway inflammation, oxidative stress, and airway remodeling than Rhy. Rhy-SLNs mitigated the asthma progression via the upregulation of SOCS1 by suppressing the p38 signaling pathway.

### Physicochemical characterization of Rhy-SLNs

**Figure 2.**
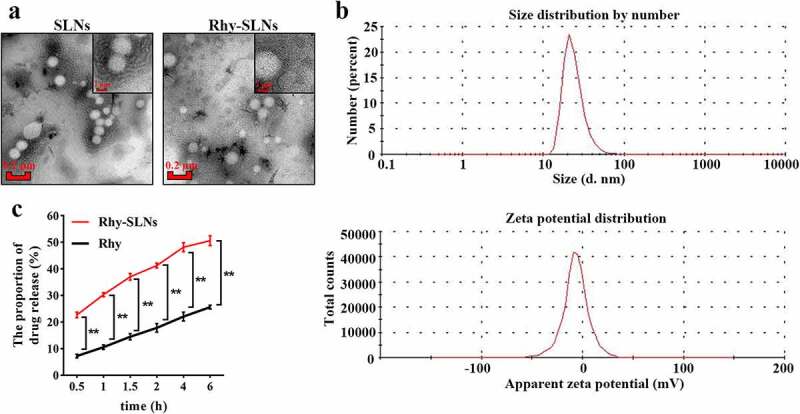
Physicochemical characterization of Rhy-SLNs. a, transmission electron microscopy images of Rhy-SLNs and SLNs. b, the size distribution and zeta potential distribution of Rhy-SLNs. c, the release of Rhy from SLNs. n = 3 in each group. *p < 0.05, **p < 0.01. Rhy, rhynchophylline; Rhy-SLNs, rhynchophylline-solid lipid nanoparticles.

Figure 2(a) shows the transmission electron microscope images of the SLNs with or without Rhy. Both SLNs had predominantly spherical shapes with dense content. Then, the mean particle size of Rhy-SLNs was found to be 62.06 ± 1.62 nm with a polymer dispersity index of 0.25 ± 0.01 and its zeta potential value was obtained as −6.53 ± 0.04 mV by using mastersizer (Figure 2(b)). The entrapment efficiency and drug-loading capacity were calculated as 82.6 ± 1.8% and 3.52 ± 0.20%. Furthermore, the release pattern of Rhy in SLNs or PBS had been revealed in Figure 2(c). Initially, Rhy-SLNs exhibited burst effects with about 41.28 ± 0.91% drug released in the first 2 hours, which might result from the release of Rhy adsorbed on the surface of nanoparticles. Subsequently, the release of Rhy in SLNs was much higher than the drug release in PBS at each time point. Drug release was found to be 50.62 ± 1.85% within 6 hours, and the sustain release might be owing to the spread of drug molecules through the lipid matrix of SLNs.

To better understand the release process, five fitting models (zero-order model, first-order model, Higuchi model, Ritger-Peppas model, and Weibull model) were applied to simulate the release kinetic characteristics of Rhy and Rhy-SLNs, respectively. As shown in Table 1, compared with the four kinds of kinetic equations, both Rhy and Rhy-SLNs had the highest R-squared value in the Weibull model kinetic equation. The value of the exponent β is an indicator of the mechanism of transport of a drug through the matrix [25]. According to the Weibull model, the value of β was 0.4966 or 0.49122 (β ≤ 0.75), suggesting the Fickian diffusion mechanism [34].

**Table 1.** Kinetic models applied to estimate obtained results

	Model	Equation	R^2^
Rhy	Zero-order kinetics	Q = 2.1464 t + 10.534	0.8808
	First-order kinetics	Ln (100-Q) = −0.0268 t + 4.4958	0.9009
Rhy-SLNs	Higuchi kinetics Ritger-Peppas kinetics Weibull kinetics Zero-order kinetics First-order kinetics Higuchi kinetics Ritger-Peppas kinetics Weibull kinetics	Q = 8.8095 t^1/2^ + 3.1057 LnQ = 0.4545 Lnt + 2.418 Ln{Ln[100/(100-Q)]} = 0.4996 Lnt – 2.124 Q = 4.5794 t + 27.01 Ln (100-Q) = −0.0759 t + 4.297 Q = 15.714 t^1/2^ + 15.423 LnQ = 0.3254 Lnt + 3.4194 Ln{Ln[100/(100-Q)]} = 0.4122 Lnt – 1	0.9629 0.9718 0.9774 (β = 0.4996) 0.8113 0.853 0.9146 0.9546 0.9663 (β = 0.4122)

R^2^ is the squared correlation coefficient.

### Rhy-SLNs attenuated the OVA-induced airway inflammation of murine experimental allergic asthma

**Figure 3.**
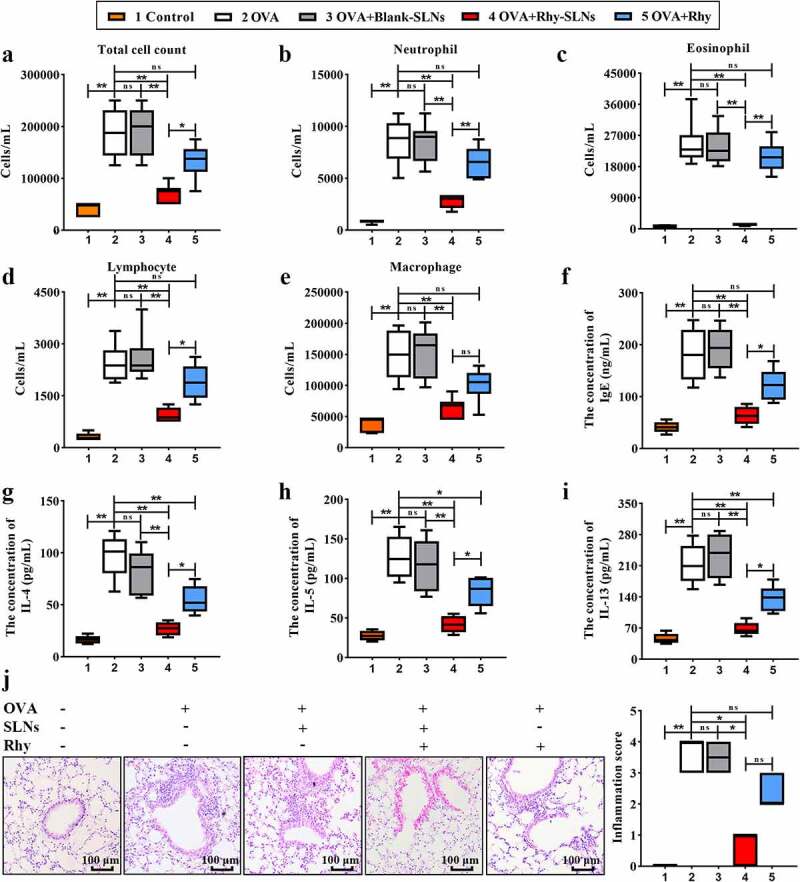
Rhy-SLNs attenuated the OVA-induced airway inflammation of murine experimental asthma. Mice were subcutaneously injected with 20 μg OVA mixed with 1 mg aluminum hydroxide on days 0, 14, 28, and 42 and administrated aerosolized 1% OVA (w/v) by inhalation from day 21 to day 42. Mice were intraperitoneally injected with 20 mg/kg Rhy-SLNs or 20 mg/kg Rhy at 1 hour before the airway challenge with OVA. a, the count of total inflammatory cells. b-e, the count of neutrophil, eosinophil, lymphocyte, and macrophage. f-i, the concentration of IgE, IL-4, IL-5, and IL-13. J, the representative images of hematoxylin-eosin-stained airway in lung tissues with inflammatory score. n = 6 in each group. *p < 0.05, **p < 0.01. IL, interleukin; OVA, ovalbumin; Rhy, rhynchophylline; Rhy-SLNs, rhynchophylline-solid lipid nanoparticles

Allergic asthma was a complex inflammatory disease characterized by airway inflammatory disorder [35]. To uncover the anti-inflammatory function of Rhy-SLNs, the count of inflammatory cells and the secretion of inflammatory factors were detected. The morphological observations were in line with the results of BALF cell-counting. Compared to Rhy, Rhy-SLNs significantly reversed the increased count of total cells, neutrophil, eosinophil, lymphocyte, and macrophage induced by OVA (Figure 3(a-e)). Moreover, Rhy-SLNs exerted a stronger inhibitory effect than Rhy on the elevated concentration of IgE in serum, as well as the increased concentration of IL-4, IL-5, and IL-13 in BALF induced by OVA (Figure 3(f-i)). Similarly, the same results of inflammatory infiltration in bronchus were obtained by hematoxylin-eosin staining (Figure 3(j)). Although there was no significant difference of inflammation score between OVA+Rhy-SLNs and OVA+Rhy groups, a tendency showed that Rhy-SLNs had better anti-inflammation effects of murine experimental asthma than Rhy.

### Rhy-SLNs alleviated the OVA-induced oxidative stress of murine experimental allergic asthma

**Figure 4.**
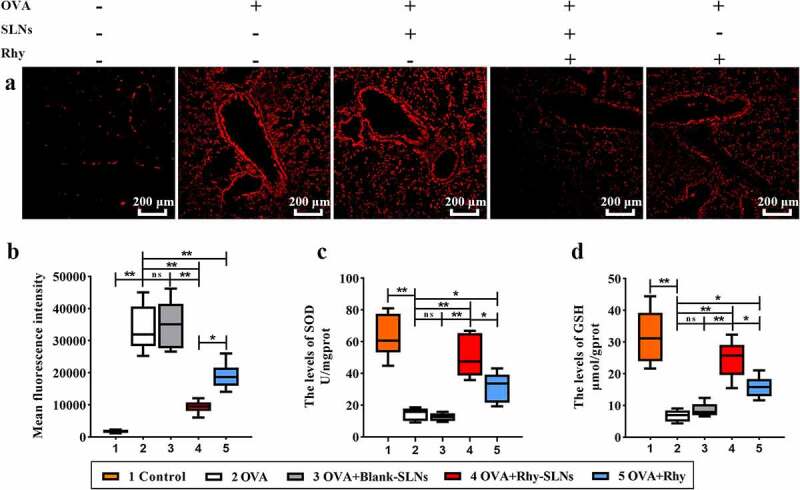
Rhy-SLNs alleviated the OVA-induced oxidative stress of murine experimental asthma. Mice were subcutaneously injected with 20 μg OVA mixed with 1 mg aluminum hydroxide on days 0, 14, 28, and 42 and administrated aerosolized 1% OVA (w/v) by inhalation from day 21 to day 42. Mice were intraperitoneally injected with 20 mg/kg Rhy-SLNs or 20 mg/kg Rhy at one hour before the airway challenge with OVA. a, the representative images of ROS probe-stained airway in lung tissues. b, the mean fluorescence intensity. c and d, the levels of SOD and GSH in lung tissues. n = 6 in each group. *p < 0.05, **p < 0.01. GSH, glutathione; OVA, ovalbumin; Rhy, rhynchophylline; Rhy-SLNs, rhynchophylline-solid lipid nanoparticles; ROS, reactive oxygen species; SOD, superoxide dismutase

Oxidative stress is a clinical and pathological hallmark in asthma, and it acts as a vital part in the allergic asthma progression [36]. Thus, we explored the effects of Rhy-SLNs on the oxidative stress of murine experimental asthma. As shown in Figure 4(a,b), Rhy-SLN more markedly reduced the OVA-induced increase in fluorescence intensity around the bronchus in lung tissues than Rhy. Moreover, Rhy-SLNs played a better role in recovering the OVA-induced decline of the SOD and GSH levels in lung tissues (Figure 4(c,d)). These results indicate that encapsulation of Rhy with SLNs allows them to be more efficacious to alleviate the OVA-induced oxidative stress of murine experimental allergic asthma than Rhy.

### Rhy-SLNs ameliorated the OVA-induced airway remodeling of murine experimental allergic asthma

**Figure 5.**
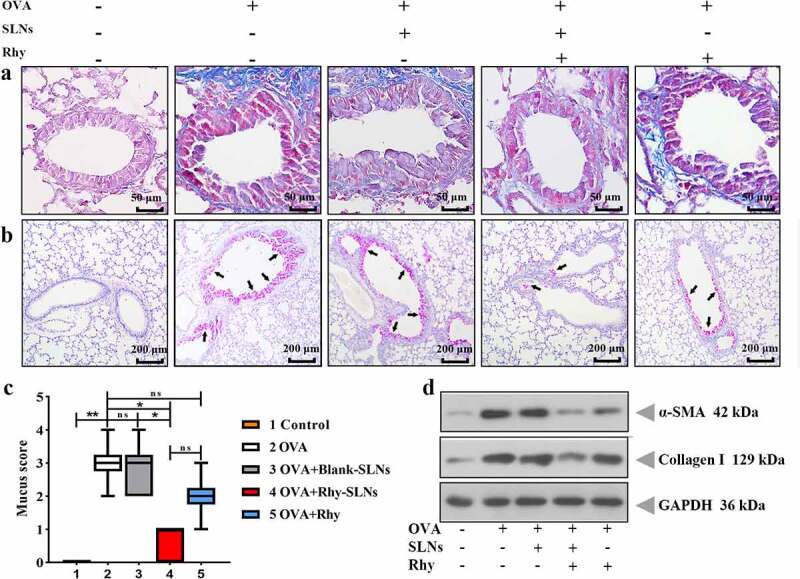
Rhy-SLNs ameliorated the OVA-induced airway remodeling of murine experimental asthma. Mice were subcutaneously injected with 20 μg OVA mixed with 1 mg aluminum hydroxide on days 0, 14, 28, and 42 and administrated aerosolized 1% OVA (w/v) by inhalation from day 21 to day 42. Mice were intraperitoneally injected with 20 mg/kg Rhy-SLNs or 20 mg/kg Rhy at one hour before the airway challenge with OVA. a, the representative images of Masson-stained airway in lung tissues. b and c, the representative images of periodic acid-Schiff-stained goblet cells of airway in lung tissues, which were quantified with the mucus score. Black arrows represented the goblet cells. d, the protein levels of α-SMA and collagen I. n = 6 in each group. *p < 0.05, **p < 0.01. OVA, ovalbumin; Rhy, rhynchophylline; Rhy-SLNs, rhynchophylline-solid lipid nanoparticles; α-SMA, alpha-smooth muscle actin

Airway remodeling is a typical structural change in allergic asthma including the deposition of collagen and the mucus gland hyperplasia, which is assessed by the number of mucous-containing goblet cells [8]. As shown in Figure 5(a), the area of OVA-induced peribronchial deposition of collagen assessed by Masson’s trichrome in the Rhy-SLNs-exposed group was decreased by Rhy-SLNs. Their suppressed effects were much better than that of Rhy group. Next, periodic acid-Schiff staining demonstrated that the increased number of goblet cells induced by OVA was reduced by the treatment of Rhy-SLNs (Figure 5(b)). Although there was no significant difference in the mucus score between Rhy-SLNs and Rhy groups, a tendency indicated that Rhy-SLNs possessed a better function in inhibiting the OVA-induced gland hyperplasia than Rhy (Figure 5(c)). Furthermore, to deeply understand the suppressed effects of Rhy-SLNs on OVA-induced airway remodeling, the expression of its markers including α-SMA and collagen I was evaluated. Rhy-SLNs weakened the elevated protein levels of α-SMA and collagen I induced by OVA (Figure 5(d)). The data indicated that administration of Rhy-SLNs was more effective than Rhy to ameliorate the OVA-induced airway remodeling of murine experimental asthma.

### Rhy-SLNs protected airway from OVA-induced damage through the upregulation of SOCS1 by repressing the p38 signaling pathway

**Figure 6.**
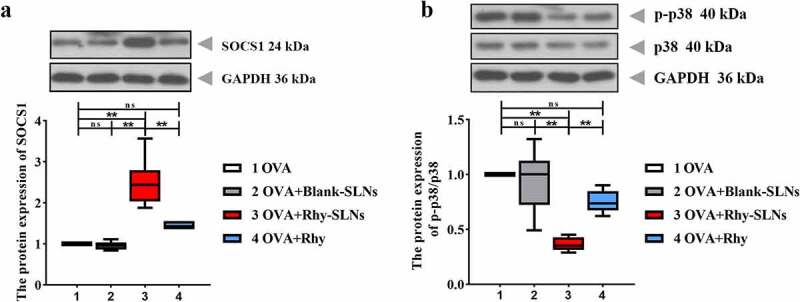
Rhy-SLNs protected airway from OVA-induced damage through the upregulation of SOCS1 by repressing the p38 signaling pathway. Mice were subcutaneously injected with 20 μg OVA mixed with 1 mg aluminum hydroxide on days 0, 14, 28, and 42 and administrated aerosolized 1% OVA (w/v) by inhalation from day 21 to day 42. Mice were intraperitoneally injected with 20 mg/kg Rhy-SLNs or 20 mg/kg Rhy at one hour before the airway challenge with OVA. a and b, western blot bands of SOCS1, p-p38, and p38. n = 6 in each group. *p < 0.05, **p < 0.01. OVA, ovalbumin; Rhy, rhynchophylline; Rhy-SLNs, rhynchophylline-solid lipid nanoparticles; SOCS1, suppressor of cytokine signaling 1

**Figure 7.**
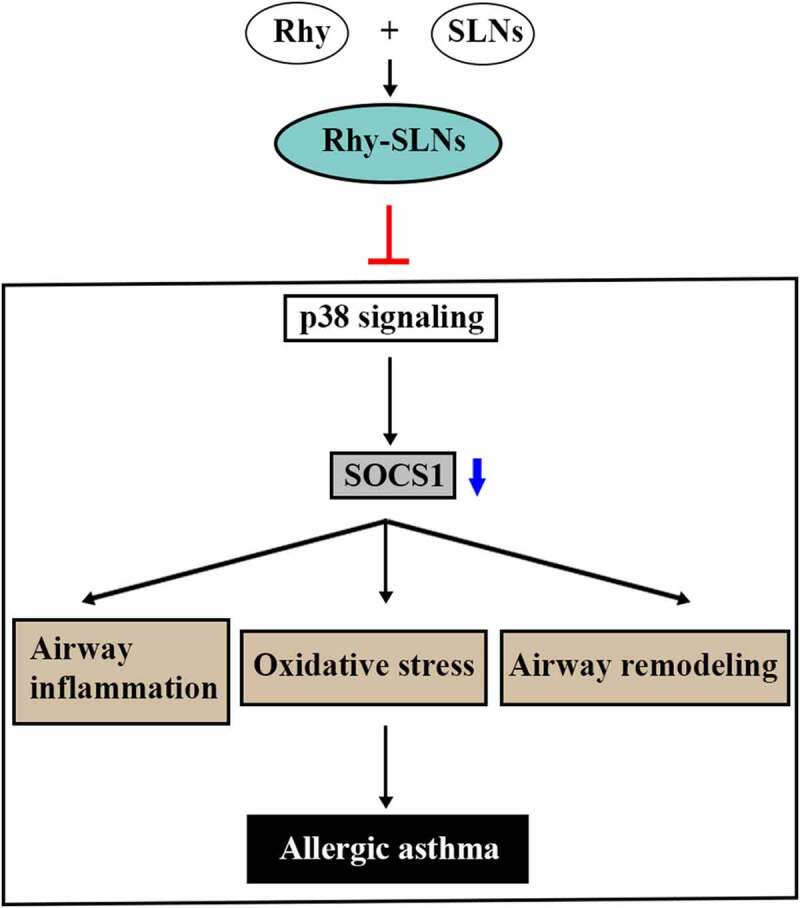
The mechanism of the inhibitory effects of Rhy-SLNs on allergic asthma. Rhy, rhynchophylline; Rhy-SLNs, rhynchophylline-solid lipid nanoparticles; SOCS1, suppressor of cytokine signaling 1

We further explore the underlying mechanism that drives the effects of Rhy-SLNs on asthma progression. SOCS1 is a negative regulator of IL-4-dependent pathway, resulting in the regulation of IgE, IL-4 and IL-13 levels, as well as the eosinophilic mucosal inflammation in asthma [37]. Based on the results of Rhy-SLNs on OVA-induced inflammation, we speculated that SOCS1 might play a role in the anti-asthma function of Rhy-SLNs. Herein, we found that Rhy-SLNs increased the SOCS1 expression of lung tissues induced by OVA (Figure 6(a)). In our previous study, we found that Rhy repressed the p38 MAPK pathway in lung tissues of murine experimental asthma [17]. Herein, the similar results were observed. The relative expression of p-p38 was reduced by Rhy-SLNs during the onset of allergic asthma (Figure 6(b)). These results suggested that Rhy-SLNs protected airway from OVA-induced damage through the upregulation of SOCS1 by repressing the p38 signaling pathway (Figure 7).

## Discussion

In the current study, Rhy-SLNs revealed a mean particle size of 62.06 ± 1.62 nm with a zeta potential value of −6.53 ± 0.04 mV and 82.6 ± 1.8% drug entrapment efficiency. The release curve of Rhy-SLNs was much higher than the drug released in phosphate buffer saline at 0, 1, 1.5, 2, 4, or 6 h. Next, we found that Rhy-SLNs exerted better effects on repressing airway inflammation, oxidative stress, and airway remodeling than that of Rhy. Furthermore, Rhy-SLNs protected airway from OVA-induced damage through the upregulation of SOCS1 by suppressing the p38 signaling pathway.

Rhy is the most abundant and major alkaloid extracted from *Uncaria rhynchophylla*, and it approximately accounts for 1/3-1/2 of the total alkaloid in *Uncaria rhynchophylla* [12]. Rhy is widely used for the treatment of cerebral disorders and cardiovascular diseases by exerting anti-inflammatory and vasodilator functions [12]. However, the low bioavailability and low aqueous solubility of Rhy limit its bioavailability and clinical efficacy. Looking for a drug carrier system for Rhy that can solve these problems might be a great policy for the treatment of asthma. SLNs are excellent drug-delivery carrier that possess great biocompatibility and controlled release of drugs [19]. Scholars have testified that curcumin loaded in SLNs effectively improves its drug bioavailability and represses the airway hyperresponsiveness and inflammatory infiltration in the treatment of asthma [20]. Gaber has clarified that the nanosized particle of lovastatin effectively enhances its solubility, dissolution rate and in vivo bioavailability [38]. These studies suggest the potential of SLNs on the improvement of drug therapeutic efficacy as a drug-delivery carrier. In this work, we encapsulated Rhy with SLNs by solvent injection method. Since the physical stability of nanoparticles is affected by their physicochemical properties, it is critical to assess their physicochemical parameters. Herein, the size distribution and surface charge were evaluated first. We found that Rhy-SLNs were spherical with rough surface. The mean particle size of Rhy-SLNs was found to be 62.06 ± 1.62 nm with a polymer dispersity index of 0.25 ± 0.01. It has been reported that the surface charge of nanoparticles could affect the stability of the nanoparticle [39]. The results in this study demonstrated that the zeta potential value of Rhy-SLNs was obtained as −6.53 ± 0.04 mV, which might prevent nanoparticle aggregation and keep their stability.

Allergic asthma is a common chronic disease that can be sensitized or worsened by allergen. Allergic asthma is linked with variable expiratory airflow and is characterized by the airway inflammation, bronchoconstriction, oxidative stress, collagen deposition, and mucus [4]. Our previous study has demonstrated that 40 mg/kg Rhy treatment by gavage protects murine airway against OVA-induced damages of allergic asthma by mitigating inflammation, airway remodeling, and oxidative stress [16,17]. In the current study, 20 mg/kg Rhy-SLNs or Rhy were subjected to OVA-induced mice to compare their therapeutic effects. We found that encapsulation of Rhy with SLNs allowed them more efficacious to alleviate the OVA-induced airway inflammation, oxidative stress, collagen deposition and mucus gland hyperplasia of murine experimental asthma than Rhy. These results indicated that the dosage of Rhy-SLNs was less than that of Rhy to achieve the same therapeutic effects on asthma.

Significantly, the way of the Rhy-SLNs administration in this work was intraperitoneal injection rather than nasal inhalation. After the inhaled administration, only a fraction of the dose is deposited in the lungs, and the rest is swallowed [11]. It has been reported that the administration of SLN-loaded drug by intraperitoneal injection could increase the tissue distribution of drug on lung [40]. The deposition of drug in lung and the further absorption act as a vital part in the treatment of asthma. Next, both the topically deposited drugs in lung and the drugs absorbed from the gastrointestinal tract will eventually enter the systemic circulation [11]. The drug in the systemic circulation will ultimately appear in the lung tissues again [11]. While drugs are absorbed quickly by intraperitoneal injection due to the large surface area of peritoneum, resulting in the quick entrance of drugs into systemic circulation [41]. Moreover, SLN-loaded drug for the treatment of asthma by intraperitoneal injection has been reported to exert excellent effects on alleviating asthma [20]. Thus, we administrated mice with Rhy or Rhy-SLNs by intraperitoneal injection in this work.

SOCS is a family of negative feedback regulators of inflammation-related signaling pathway [42]. Eight members of SOCS family have been identified including SOCS1-7 and cytokine-inducible SH2-containing protein [42]. SOCS1 is the first member of SOCS family to be discovered [42]. Accumulating evidence has testified that SOSC1 is downregulated in the airways of severe asthmatics than moderate/mild asthmatics and is linked with airway Th2 inflammation [43]. Lee et al. have found that SOCS1 is a negative regulator of IL-4-dependent pathway, resulting in the regulation of IgE levels along with IL-4 and IL-13 introduction, as well as the eosinophilic mucosal inflammation involved in asthma [37]. These studies suggest that SOCS1 acts as a vital part in the inflammatory progression of asthma. In this work, we found that Rhy-SLNs significantly elevated the protein levels of SOCS1 of lung tissues in experimental asthma. In combined with the results of Rhy-SLNs on OVA-induced airway inflammation, we concluded that the protective role of Rhy-SLNs against asthma might be meditated by the upregulation of SOCS1.

All the main triggers of asthma (such as aeroallergens, airborne pollutant, and viral infections) could activate the p38 subfamily of MAPK inside the respiratory tract [44]. p38 is able to induce the differentiation and activation of Th2 in asthma, resulting in the facilitated release of cytokines (IL-4, IL-5, and IL-13) secreted by Th2 cells [45]. Santana et al. [46] have reported that the alleviation of asthma inflammation is mediated by the suppression of JNK, p38, and ERK1/2 components of MAPK pathway. Similarly, in our previous study, we have found that Rhy attenuates asthma via inhibiting the OVA-induced hyperplasia of airway smooth muscle cells, the recruitment of eosinophils in BALF by repressing the p38 MAPK signaling pathway. Herein, the similar results were obtained. We found that the relative expression of p-p38 was decreased by Rhy-SLNs during the onset of allergic asthma. Moreover, accumulating evidence has shown that the specific inhibition of p38 and JNK is required for inducing SOCS1 expression, resulting in the repression of *Chlamydia muridarum*-induced inflammatory responses [47]. Ahemed et al. [48] have confirmed that the treatment of R9-SOCS1-KIR leads to the inhibition of p-p38 pathways in corneal uveitis. These studies indicate that the SOCS1 expression might be associated with p38 pathway in inflammatory responses. Based on the results of Rhy-SLNs on the regulation of SOCS1 levels, we concluded that Rhy-SLNs protected airway from OVA-induced damages through the upregulation of SOCS1 by repressing the p38 signaling pathway.

Our work has some limitations. First, we found that the release curve of Rhy-SLNs was much higher than the drug released in PBS in vitro. However, the in vivo release of Rhy-SLNs and Rhy has not been studied. Second, it will be more persuasive to rescue the SOCS1 and block the p38 signaling for the mechanism of the anti-asthma effects of Rhy-SLNs. Future investigation should take these short comings into account. Moreover, we will further explore the precise mechanism on why Rhy-SLNs protected airway from OVA-induced damage through the upregulation of SOCS1 by repressing the p38 signaling pathway in the future. Next, the stability as well as Rhy incorporation and expulsion rates of Rhy-SLN need further improvement. We believe that Rhy-SLNs might be an effectual drug in the treatment of allergic asthma.

## Conclusion

Rhy-SLNs exerted better effects on repressing airway inflammation, oxidative stress, and airway remodeling than Rhy. Rhy-SLNs mitigated the asthma progression via the upregulation of SOCS1 by suppressing the p38 signaling pathway.

## Data Availability

The data of this work is available on request to the corresponding author.
